# The mediating role of perceived stress between the job demand-control-support model and occupational fatigue among operating room nurses: a multi-Center cross-sectional study

**DOI:** 10.3389/fpubh.2026.1947875

**Published:** 2026-10-27

**Authors:** Wenjing Qian, Liping Wu, Xiaoning Wang

**Affiliations:** Department of Nursing, Ruijin Hospital, Shanghai Jiao Tong University School of Medicine, Shanghai, China

**Keywords:** cross-sectional study, JDCS model, occupational fatigue, operating room nurses, perceived stress

## Abstract

**Objective:**

Operating room nurses work long-term in high-intensity, high-risk environments and suffer from a high prevalence of occupational fatigue, which seriously affects their physical and mental health as well as patient safety. Based on the Job Demand-Control-Support (JDCS) model, this study aimed to examine the mediating role of perceived stress between the dimensions of the JDCS model and occupational fatigue—including acute fatigue, chronic fatigue, and inter-shift recovery—among operating room nurses.

**Methods:**

This multi-center cross-sectional study was conducted from August to September 2026, enrolling 1,122 operating room nurses from 9 tertiary Grade-A hospitals in eastern, central, and western China. A general information questionnaire, the Occupational Fatigue Exhaustion Recovery Scale, the Perceived Stress Scale (PSS-10), and the Job Content Questionnaire (JCQ) were administered. SPSS 25.0 was used for descriptive statistics, difference analysis, correlation analysis, and hierarchical multiple regression; AMOS 21.0 was used to construct a structural equation model, and the bias-corrected Bootstrap method was employed to test mediating effects.

**Results:**

The scores of acute fatigue, chronic fatigue, and inter-shift recovery among operating room nurses were 45.55 ± 18.77, 44.13 ± 17.89, and 56.27 ± 17.83, respectively, indicating an overall moderate level of occupational fatigue. The perceived stress score was 21.01 ± 5.24; job demand was 36.44 ± 4.80; job control was 71.54 ± 18.07; and social support was 24.02 ± 6.61. Hierarchical regression showed that, after controlling for demographic and work characteristics, job demand, job control, and social support remained independent correlates of all three outcomes (acute fatigue model *R*^2^ = 0.460, chronic fatigue *R*^2^ = 0.305, inter-shift recovery *R*^2^ = 0.353). The structural equation model showed that job demand positively predicted perceived stress (*β* = 0.182, *p* < 0.001), while job control (*β* = −0.251, *p* = 0.033) and social support (*β* = −0.168, *p* = 0.040) negatively predicted perceived stress. Perceived stress positively predicted acute fatigue (*β* = 0.336, *p* < 0.001) and chronic fatigue (*β* = 0.209, *p* < 0.001), and negatively predicted inter-shift recovery (*β* = −0.134, *p* = 0.029). Bootstrap tests indicated that perceived stress played a significant partial mediating role between job demand, job control, and social support and the outcomes of acute fatigue, chronic fatigue, and inter-shift recovery, although the indirect effects were small in magnitude (proportion mediated 3.41–17.35%).

**Conclusion:**

Occupational fatigue among operating room nurses in 9 tertiary Grade-A hospitals is at a moderate level, and inter-shift recovery needs improvement. Perceived stress acted as a mediating variable linking JDCS dimensions to occupational fatigue, although the mediating effects were modest in magnitude. Nursing managers can improve occupational fatigue and recovery among operating room nurses by reducing unreasonable job demands, enhancing job control, strengthening social support, and alleviating perceived stress.

## Introduction

The operating room is a core department for surgical treatment and emergency and critical care rescue, and its nursing quality is directly related to patient safety. Operating room nurses are a group working in a specific environment and engaged in a specialized nursing profession. Owing to the high workload, high stress, and high-risk nature of operating room nursing, they are facing the threat of high levels of physical and psychological fatigue (1). In recent years, multiple domestic and international surveys have shown that the prevalence of occupational fatigue among operating room nurses is as high as 71 to 94.8% (2, 3). Occupational fatigue not only leads to increased absenteeism and turnover among nurses but also impairs their attention, judgment, and emergency response capabilities, increasing the risk of nursing errors and, in severe cases, endangering patient safety (4, 5). Taking occupational fatigue as the entry point to explore its influencing factors and mechanisms is of great theoretical and practical significance for alleviating nurse fatigue, promoting physical and mental health, stabilizing the nursing workforce, and improving the quality of operating room nursing.

Nurse occupational fatigue refers to a multidimensional state in which insufficient recovery—caused by exposure to excessive demands and stress in work tasks, environment, and scheduling—interferes with physical, cognitive, and work functioning (6). According to duration, it is classified into acute fatigue, delayed fatigue, and chronic fatigue. Acute fatigue reflects immediate physical and mental exhaustion after a single shift; chronic fatigue reflects long-term cumulative exhaustion; and inter-shift recovery reflects an individual’s ability to restore energy and physical strength between shifts, representing a restorative outcome (7). Previous studies have suggested that job stress, working hours, shift patterns, job demand, job control, and social support are all important factors influencing occupational fatigue among nurses (8–11). However, most studies have focused on only a single or a few variables, lacking an integrated theoretical framework to explain the comprehensive mechanisms among these factors.

The Job Demand-Control-Support (JDCS) model was proposed by Johnson. Its core variables include job demand, job control, and social support, and it posits that these three factors influence job stress through main effects and buffering effects, which in turn affect occupational health outcomes (12). The model holds that job demand, job control, and social support not only exert main effects on job stress respectively, but the latter two can also buffer the stress caused by job demand. This study focused on the main-effect pathway of the JDCS model, examining the direct associations of job demand, job control, and social support with perceived stress and different fatigue and recovery outcomes, as well as the potential mediating role of perceived stress therein. The buffering (moderating) effect was not within the scope of this study and will be examined in future research.

In this study, the mediating variable was labeled perceived stress, which refers to an individual’s appraisal of the extent to which situations in one’s life are perceived as stressful. Conceptually, the mediator of interest corresponds to the stress response triggered when job demands exceed the working capacity of operating room nurses (13). Perceived stress was used to represent the job stress experienced by operating room nurses, and the limitations of this operationalization are acknowledged. Studies have shown that job stress is a significant predictor of occupational fatigue among nurses (14, 15), and the higher the stress level, the more severe the fatigue perceived by nurses (16). Steege et al. stated that effective management of work-related stressors is a core strategy for reducing occupational fatigue (17). For operating room nurses, factors such as insufficient staffing, excessive workload, frequent shift rotation, high professional skill requirements, and inadequate support can all induce and accumulate job stress (18).

The JDCS model integrates external organizational stimuli with individual stress responses, viewing job stress as a dynamic process involving independent variables, dependent variables, and mediating and buffering variables. Job control refers to the degree of control nurses have over work content, pace, and decision-making processes, which directly affects job satisfaction and fatigue levels; long-term high stress and overloaded work can weaken job control and exacerbate occupational fatigue (19). Domestic studies have shown significant associations among stressors, fatigue, and sense of control (16), highlighting the important role of job control in the regulation of occupational fatigue and stress. As an important environmental factor, social support mainly comes from superiors and colleagues, taking the forms of emotional support, collaborative assistance, professional guidance, and practical feedback (20, 21). Studies have found that fatigue status is negatively correlated with social support and positively correlated with job stress; job stress, physical condition, and job stress are key predictors of fatigue (22). Zhu Huimin et al. found that social utilization, social support, and the management of job stress are negatively correlated with three dimensions—interpersonal relationships, time allocation and workload, and nursing professional work—as well as with occupational fatigue (23). Adriaenssens et al. pointed out that reducing job demand, enhancing sense of control, and strengthening social support can effectively improve occupational fatigue (24).

However, for the specific population of operating room nurses, the following research gaps remain. First, at the empirical level, existing studies on occupational fatigue among nurses have mostly focused on groups such as emergency, intensive care, and general ward nurses; empirical evidence for operating room nurses—a special group with high work intensity, high technical requirements, and uncontrollable pace—remains limited, and most studies have included only a single or a few predictor variables. Second, at the theoretical level, although the JDCS model has been widely applied in occupational health research, there is a lack of systematic examination under a mediating framework of how the dimensions of the JDCS model influence fatigue outcomes across different time dimensions (acute fatigue, chronic fatigue, and inter-shift recovery) through perceived stress; it is also unclear whether the role of perceived stress as a mediator varies by outcome. Third, at the practical level, operating rooms in Chinese tertiary Grade-A hospitals generally share organizational characteristics such as large surgical volumes, tight scheduling, and relatively insufficient staffing, yet relevant research evidence is insufficient, making it difficult to provide targeted evidence for local nursing management. Based on these gaps, this study drew on the JDCS model, took operating room nurses from Chinese tertiary Grade-A hospitals as the research subjects, used job demand, job control, and social support as antecedent variables, perceived stress as a mediating variable, and acute fatigue, chronic fatigue, and inter-shift recovery as outcome variables, to construct and test a model of the influencing mechanisms of occupational fatigue among operating room nurses, with the aim of providing empirical evidence for stratified and precise nursing management interventions.

Based on the JDCS main-effect hypothesis and previous studies, this study proposed directionally explicit hypotheses for each outcome variable:

*H1*: Job demand is associated with fatigue and recovery outcomes; *H1b*: Job demand positively predicts chronic fatigue; *H1c*: Job demand negatively predicts inter-shift recovery.

*H2*: Job control is associated with fatigue and recovery outcomes; *H2a*: Job control negatively predicts acute fatigue; *H2b*: Job control negatively predicts chronic fatigue; *H2c*: Job control positively predicts inter-shift recovery.

*H3*: Social support is associated with fatigue and recovery outcomes; *H3a*: Social support negatively predicts acute fatigue; *H3b*: Social support negatively predicts chronic fatigue; H3c: Social support positively predicts inter-shift recovery.

*H4*: Perceived stress is associated with fatigue and recovery outcomes; *H4a*: Perceived stress positively predicts acute fatigue; *H4b*: Perceived stress positively predicts chronic fatigue; *H4c*: Perceived stress negatively predicts inter-shift recovery.

*H5*: The dimensions of the JDCS model are associated with perceived stress; *H5a*: Job demand positively predicts perceived stress; *H5b*: Job control negatively predicts perceived stress. *H5c*: Social support negatively predicts perceived stress.

*H6*: Perceived stress mediates the relationship between job demand and fatigue and recovery outcomes (H6a: acute fatigue; H6b: chronic fatigue; H6c: inter-shift recovery).

*H7*: Perceived stress mediates the relationship between job control and fatigue and recovery outcomes (H7a: acute fatigue; H7b: chronic fatigue; H7c: inter-shift recovery).

*H8*: Perceived stress mediates the relationship between social support and fatigue and recovery outcomes (H8a: acute fatigue; H8b: chronic fatigue; H8c: inter-shift recovery).

## Materials and methods

### Study design

This study was a multi-center cross-sectional survey.

### Sample and data collection

This study used a multi-stage mixed sampling method to select research subjects. First, according to the economic and geographical divisions of eastern, central, and western China, and to ensure regional diversity, the research team purposively selected 3 provinces, municipalities, or autonomous regions from each region, for a total of 9 provincial-level administrative units. Subsequently, within each provincial-level unit, one tertiary Grade-A hospital was purposively selected as the research site. The 9 hospitals finally included were located in Shanghai, Beijing, Zhejiang Province, Hubei Province, Jiangxi Province, Henan Province, Sichuan Province, Guangxi Zhuang Autonomous Region, and Shaanxi Province. Finally, in each selected hospital, uniformly trained investigators distributed questionnaires to all operating room nurses who met the inclusion criteria using convenience sampling. From August to September 2026, the researchers recruited all operating room nurses and invited voluntary participants to complete the survey. Inclusion criteria: (1) licensed nurses who had worked in the operating room of a tertiary Grade-A hospital in mainland China for 1 year or more; (2) voluntary participation in this study. Exclusion criteria: (1) visiting/training nurses; (2) nurses on sick or personal leave or not working in the department during the survey period.

According to sample size calculation methods, the sample size required for a structural equation model should be 5–10 times the number of variables (25). This study included a total of 47 variables, requiring 235–470 subjects; considering a 20% attrition rate, the sample size should be 294–588 subjects. A total of 1,216 questionnaires were distributed, and 1,122 valid questionnaires were recovered, meeting the sample size requirement.

### Instruments

#### General information questionnaire

Designed by the researchers, this questionnaire included sociodemographic data and work-related data. Sociodemographic data included region, gender, age, years of work experience, education, position, professional title, competency level, employment type, marital status, childbirth status, and total monthly income. Work-related data included surgical specialty, surgical level, daily surgical caseload, daily overtime duration, work pattern, shift rotation cycle, monthly number of night shifts, interval between night shifts, and daily sleep duration. To clarify operational definitions and reduce recall bias, the recall periods and calculation methods for work-related variables were uniformly specified as follows: daily surgical caseload refers to the average number of surgeries assisted per day in the month prior to the survey; daily overtime duration refers to the average number of hours exceeding standard working hours per day in the month prior to the survey; work pattern was classified into fixed day shift and rotating shift; shift rotation cycle was classified into regular, irregular, and flexible; monthly number of night shifts refers to the actual number of night shifts undertaken in the month prior to the survey (recorded as 0 for those without night shifts); interval between night shifts refers to the average number of days between two consecutive night shifts (only for those with night shifts); daily sleep duration refers to the average number of hours of sleep per day in the month prior to the survey.

#### Occupational fatigue exhaustion recovery scale

The Chinese version of the Occupational Fatigue Exhaustion Recovery Scale, translated by Fang Jinbo, was used. It comprises 3 subscales—chronic fatigue (5 items), acute fatigue (5 items), and inter-shift recovery (5 items)—with a total of 15 items (26). A 7-point Likert scale was used, with 0 indicating “strongly disagree” and 6 indicating “strongly agree.” Scores for each dimension were converted to a percentage scale; higher scores indicated more severe fatigue or better recovery. In this study, the Cronbach’s α coefficients of the 3 subscales were 0.906, 0.895, and 0.889, respectively, and the overall Cronbach’s α of the scale was 0.912.

#### Perceived stress scale-10

The Perceived Stress Scale, developed by Cohen et al. (27) and translated into Chinese by Wang Zhen et al. (28) was used to assess the participants’ global perceived stress over the past month. The scale consists of 10 items and uses a 5-point Likert scale, scored from 0 (“never”) to 4 (“always”), with a total score ranging from 0 to 40; higher scores indicate greater perceived stress. The Cronbach’s α coefficient of this scale in this study was 0.881.

#### Job content questionnaire

The Chinese version of the Job Content Questionnaire (JCQ), developed by Li Jian, was used. It consists of 3 subscales—job demand (5 items), job control (6 items for skill discretion and 3 items for decision authority), and social support (4 items for supervisor support and 4 items for coworker support)—with a total of 22 items (29). A 4-point Likert scale was used, with 1 indicating “strongly disagree” and 4 indicating “strongly agree.” The scale uses a weighted scoring rule, and the total scores of the 3 subscales, calculated according to standard formulas, range from 12 to 48, 24 to 96, and 8 to 32, respectively; higher subscale scores indicate higher job demand, stronger job control, and more social support. The Cronbach’s α coefficient of this scale in this study was 0.874.

### Data collection

The nursing departments of each hospital were contacted to obtain consent and cooperation from the nursing department and operating room head nurses. Uniformly trained research team members explained the research purpose, significance, and filling method on-site with standardized instructions during general departmental learning sessions in the operating rooms. After signing a paper informed consent form, participants completed the survey anonymously online via a QR code using the Wenjuanxing platform; all questionnaire items were set as required. Data were uniformly retrieved from the Wenjuanxing backend. To control data quality, the research team conducted logical checks on the recovered questionnaires. For questionnaires with clearly logically inconsistent responses, telephone verification was conducted through non-identifying contact information reserved at the time of questionnaire recovery, after informing the subjects and obtaining their consent. Questionnaires that still showed patterned responses, garbled responses, orclearly unrealistic responses after verification were excluded. All personal information was used only for quality verification and was deleted after data cleaning; only anonymous questionnaire numbers were retained.

### Ethical considerations

This study was approved by the Ethics Committee of Ruijin Hospital, Shanghai Jiao Tong University School of Medicine (Approval No.: 2026–273), and all procedures complied with the principles of the Declaration of Helsinki. All participants signed written informed consent forms.

### Statistical analysis

SPSS 25.0 was used for data management and statistical analysis, and AMOS 21.0 was used to construct the structural equation model. Continuous variables were described as mean ± standard deviation, and categorical data were described as frequencies and percentages. (1) Data screening: all questionnaire items were required, so there was no item-level missingness. Normality tests for continuous variables (using absolute skewness <3 and absolute kurtosis <10 as approximate normality reference criteria) and outlier checks (using mean ± 3 standard deviations as the boundary) were performed. (2) Scale psychometric testing: exploratory factor analysis was conducted for each scale to test structural validity; confirmatory factor analysis was conducted for the overall measurement model (including 9 latent variables: job demand, skill discretion, decision authority, supervisor support, coworker support, perceived stress, acute fatigue, chronic fatigue, and inter-shift recovery), reporting standardized factor loadings, composite reliability, average variance extracted, and discriminant validity. (3) Between-group difference comparisons: independent samples t-tests or one-way ANOVA were used to compare differences in fatigue and inter-shift recovery scores among populations with different demographic and work characteristics; post-hoc pairwise comparisons for multiple groups used the LSD method, corrected by the Bonferroni method. (4) Correlation analysis: Pearson correlation analysis was used to examine correlations among continuous variables. (5) Hierarchical regression: acute fatigue, chronic fatigue, and inter-shift recovery were used as dependent variables, respectively; demographic and work characteristic control variables, job demand, job control, and social support were entered hierarchically to test the incremental predictive effects of the core independent variables; multicollinearity was assessed through tolerance and variance inflation factor (VIF). (6) Structural equation model and mediating effects: a structural equation model was constructed with job demand, job control, and social support as exogenous latent variables, perceived stress as the mediating variable, and acute fatigue, chronic fatigue, and inter-shift recovery as endogenous latent variables, using maximum likelihood (ML) estimation; the bias-corrected percentile Bootstrap method (5,000 resamples) was used to estimate indirect effects and their 95% confidence intervals, with intervals not containing 0 considered significant mediating effects; model fit was comprehensively judged based on indicators such as CMIN/df (<3 acceptable), RMSEA (<0.08 acceptable), and CFI/TLI (>0.90 acceptable) (30). Path coefficients are reported as standardized estimates (β), with unstandardized estimates (B) and standard errors also provided in Table 1. A two-sided *p* < 0.05 was considered statistically significant.

**Table 1:** Path coefficients of the structural equation model.

Path	*β*	*B*	S. E.	C. R.	*p*	Hypothesis result
Perceived stress ← job control	−0.251	−4.985	2.338	−2.132	0.033	H5b supported
Perceived stress ← social support	−0.168	−1.054	0.506	−2.083	0.040	H5c supported
Perceived stress ← job demand	0.182	1.097	0.206	5.320	<0.001	H5a supported
Acute fatigue ← perceived stress	0.336	0.075	0.012	6.175	<0.001	H4a supported
Chronic fatigue ← perceived stress	0.209	0.039	0.011	3.630	<0.001	H4b supported
Inter-shift recovery ← perceived stress	−0.134	−0.027	0.012	−2.190	0.029	H4c supported
Acute fatigue ← job control	−0.844	−3.739	0.487	−7.684	<0.001	H2a supported
Chronic fatigue ← job control	−0.888	−3.304	0.434	−7.614	<0.001	H2b supported
Inter-shift recovery ← job control	0.963	3.832	0.498	7.694	<0.001	H2c supported
Acute fatigue ← job demand	0.349	0.468	0.071	6.605	<0.001	H1a supported
Chronic fatigue ← job demand	0.181	0.204	0.063	3.259	0.001	H1b supported
Inter-shift recovery ← job demand	−0.213	−0.257	0.071	−3.624	<0.001	H1c supported
Acute fatigue ← social support	−0.301	−0.421	0.121	−3.480	<0.001	H3a supported
Chronic fatigue ← social support	−0.219	−0.256	0.107	−2.405	0.016	H3b supported
Inter-shift recovery ← social support	0.194	0.243	0.121	2.010	0.044	H3c supported

β, Standardized path coefficient; B, Unstandardized path coefficient; S. E., Standard error; C. R., Critical ratio.

## Results

### General characteristics

A total of 1,216 questionnaires were recovered; after excluding 94 questionnaires with patterned responses, garbled responses, or completion time <3 min (see Figure 1), 1,122 were finally included in the analysis, with an effective recovery rate of 92.27%. The 1,122 operating room nurses had a mean age of 31.41 ± 7.35 years (range 20–59 years), monthly income of 1.13 ± 0.53 (0.1–5.0) ten thousand RMB, daily surgical caseload of 4.16 ± 2.06 (0–20) cases, and daily overtime of 2.38 ± 1.32 (0–9) hours. Regarding work pattern, 592 nurses (52.76%) worked rotating shifts, including mid-shift and night-shift personnel. Among the 592 rotating-shift nurses, 48 (8.11%) were on flexible schedules (mid-shifts and night-shifts flexibly arranged by nursing managers according to surgical conditions). Regarding monthly night shifts, 567 nurses (50.53%) had no night shifts, including day-shift and mid-shift personnel. Among the 555 nurses (49.47%) with night-shift experience, the monthly number of night shifts was 3.25 ± 1.64 (1–12).

**Figure 1:**
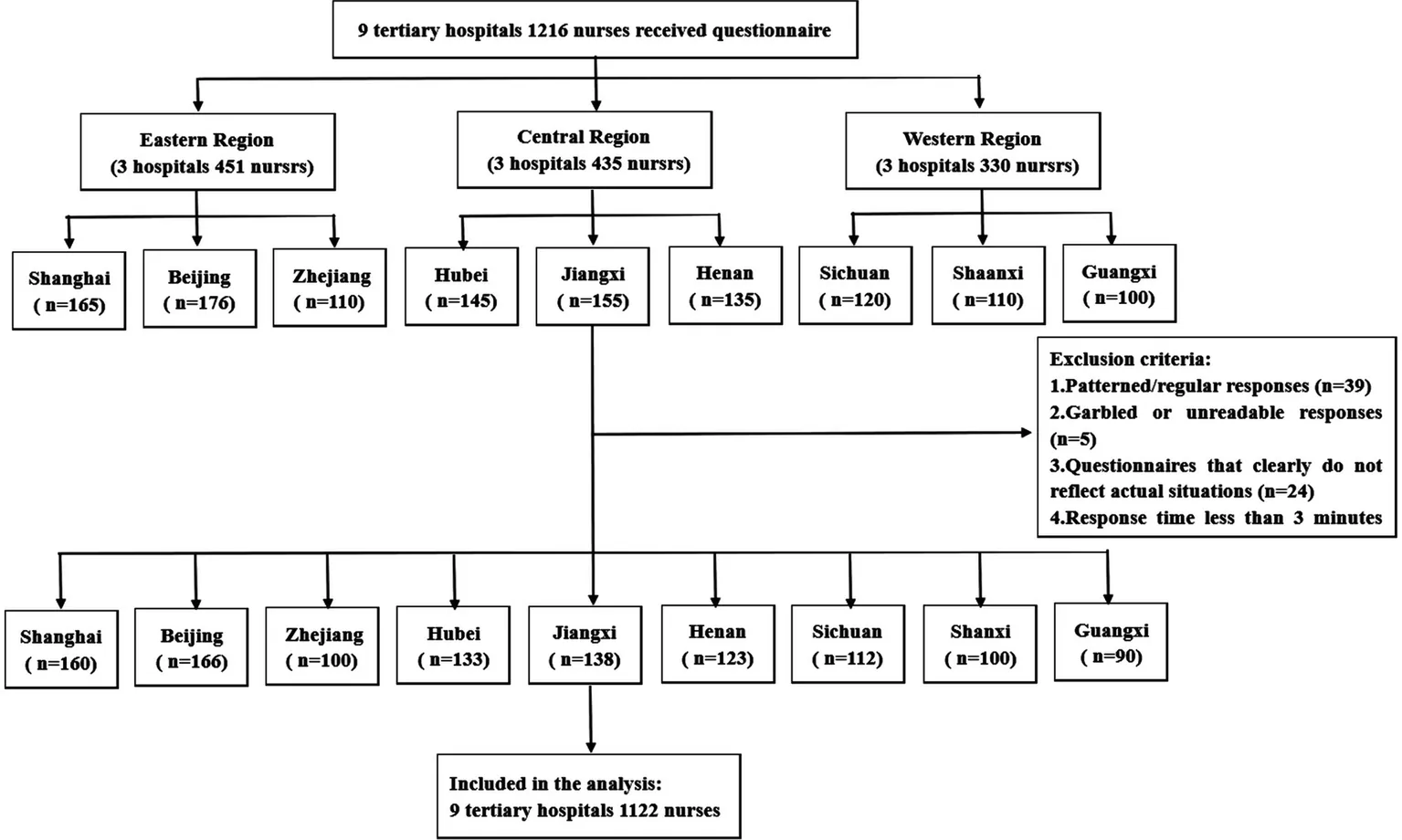
Flow chart of the selection process.

### Exploratory and confirmatory factor analysis of scales

For the Job Content Questionnaire, KMO = 0.903, Bartlett’s test of sphericity *χ*^2^ = 17710.137 (df = 231, *p* < 0.001); 5 common factors were extracted with eigenvalues >1, with a cumulative variance explanation rate of 76.099%. The 22 items clearly belonged to 5 factors—job demand, skill discretion, decision authority, supervisor support, and coworker support—consistent with the theoretical structure. For the Occupational Fatigue Exhaustion Recovery Scale, KMO = 0.948, *χ*^2^ = 11546.522 (df = 105, *p* < 0.001); 3 common factors were extracted, with a cumulative variance explanation rate of 71.167%, consistent with the theoretical dimensions. For the Perceived Stress Scale, KMO = 0.888, *χ*^2^ = 5112.966 (df = 45, *p* < 0.001), indicating the data were suitable for factor analysis.

Confirmatory factor analysis (Figure 2) showed that the overall measurement model (job content, perceived stress, occupational fatigue exhaustion recovery) had acceptable fit: CMIN/df = 2.954, RMR = 0.045, RMSEA = 0.048, GFI = 0.899, AGFI = 0.884, IFI = 0.949, TLI = 0.944, CFI = 0.948. The standardized loadings of observed items for each latent variable ranged from 0.711 to 0.930, all >0.7; the composite reliability (CR) of each dimension ranged from 0.811 to 0.927, all >0.8; the average variance extracted (AVE) ranged from 0.616 to 0.782, all >0.5, indicating good convergent validity (Table 2). Discriminant validity tests showed that the square root of AVE for each latent variable was greater than its correlation coefficient with other latent variables, indicating good discriminant validity of the scales (Table 3). At the latent level, the correlations among the three exogenous latent variables were modest: job control with social support (*r* = 0.327), job control with job demand (*r* = 0.037), and job demand with social support (*r* = −0.036); all were far below 0.8, together with the VIF values (<2) in the regression models, indicating no concern about collinearity among the exogenous latent variables.

**Figure 2:**
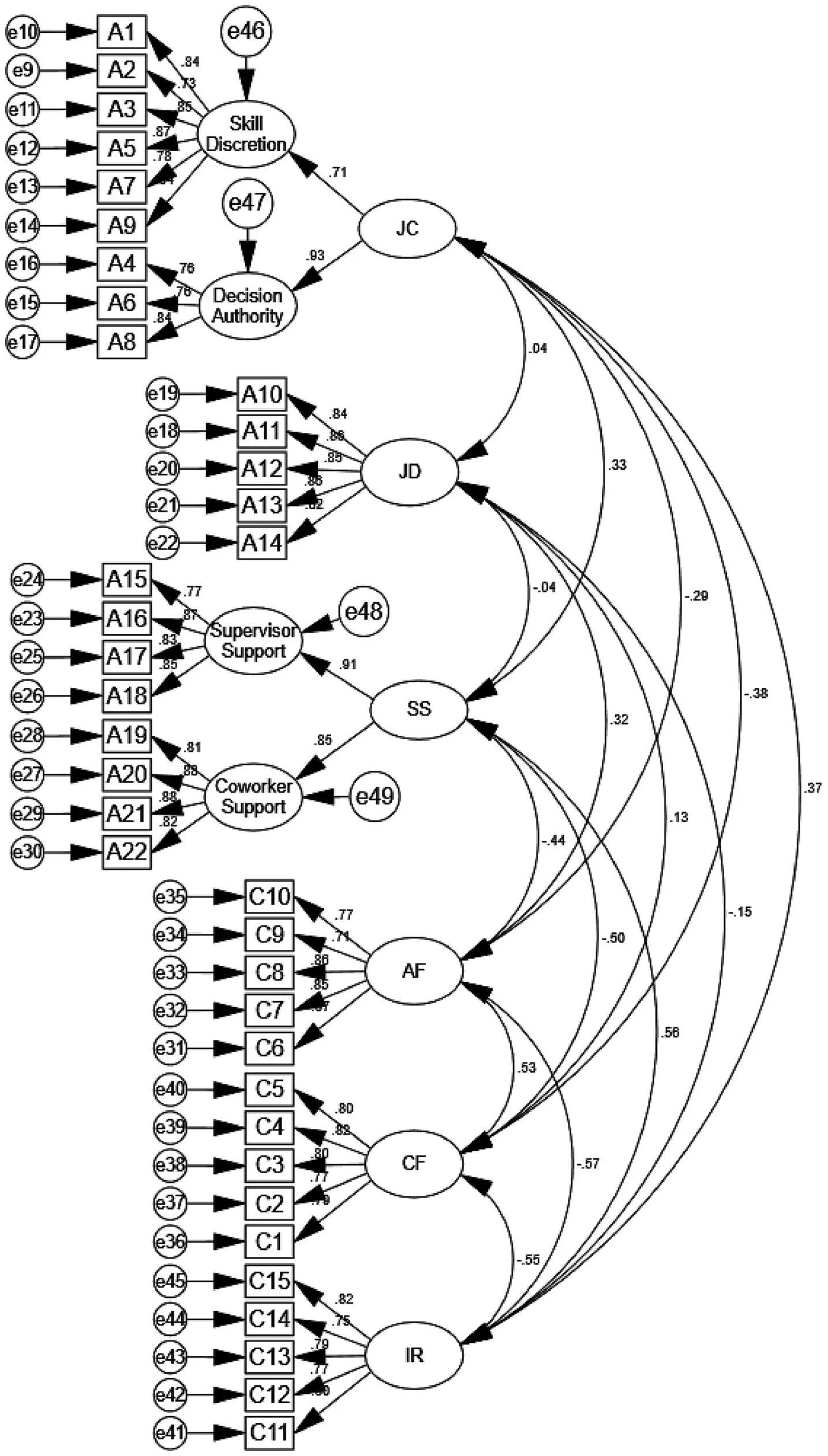
Confirmatory factor analysis (CFA) of the model.

**Table 2:** Factor loadings, composite reliability, and average variance extracted.

Latent variable	Observed dimension	Std. loading range	CR	AVE
Job control (JC)	Skill discretion, decision authority	0.711 ~ 0.930	0.811	0.685
Social support (SS)	Supervisor support, coworker support	0.854 ~ 0.914	0.878	0.782
Job demand (JD)	5 items	0.822 ~ 0.865	0.927	0.716
Skill discretion	6 items	0.733 ~ 0.870	0.924	0.670
Decision authority	3 items	0.757 ~ 0.838	0.829	0.618
Supervisor support	4 items	0.766 ~ 0.870	0.898	0.688
Coworker support	4 items	0.809 ~ 0.878	0.911	0.719
Acute Fatigue (AF)	5 items	0.713 ~ 0.868	0.907	0.661
Chronic fatigue (CF)	5 items	0.767 ~ 0.818	0.895	0.631
Inter-shift recovery (IR)	5 items	0.746 ~ 0.819	0.889	0.616

The standardized loadings of perceived stress (PS) items range from 0.7 to 0.9, with AVE>0.5; perceived stress is a unidimensional scale, and specific loadings are shown in the AMOS output.

**Table 3:** Discriminant validity.

Latent variable	SS	JC	JD	AF	CF	IR
SS	0.884					
JC	0.327	0.828				
JD	−0.036	0.037	0.846			
AF	−0.439	−0.288	0.323	0.813		
CF	−0.501	−0.382	0.133	0.527	0.794	
IR	0.555	0.374	−0.147	−0.566	−0.552	0.785

SS, Social Support; JC, Job Control; JD, Job Demand; AF, Acute Fatigue; CF, Chronic Fatigue; IR, Inter-Shift Recovery.

### Scores of occupational fatigue, perceived stress, job demand, job control, and social support

The scores of acute fatigue, chronic fatigue, and inter-shift recovery among operating room nurses were 45.55 ± 18.77, 44.13 ± 17.89, and 56.27 ± 17.83, respectively; occupational fatigue was overall at a moderate level, and inter-shift recovery still had room for improvement. The perceived stress score was 21.01 ± 5.24; job demand was 36.44 ± 4.80; job control was 71.54 ± 18.07 (including skill discretion 35.70 ± 10.33 and decision authority 35.85 ± 9.84); and social support was 24.02 ± 6.61 (including supervisor support 12.02 ± 3.56 and coworker support 12.17 ± 3.53).

### Differences in fatigue and inter-shift recovery among populations with different characteristics

There were statistically significant differences in acute fatigue, chronic fatigue, and inter-shift recovery among operating room nurses in terms of region, shift rotation cycle, and employment type (*p* < 0.05). Gender, marital status, childbirth status, years of work experience, position, professional title, and daily overtime duration showed no statistically significant differences for the three outcomes. For other demographic characteristics, see Table 4.

**Table 4:** Differences in acute fatigue, chronic fatigue, and inter-shift recovery across demographic characteristics.

Item	AF	t/F	*p*	CF	t/F	*p*	IR	t/F	*p*
Education		6.265	0.002**		3.61	0.027*		1.064	0.346
Junior college or below	42.25 ± 17.90			41.79 ± 16.50			57.57 ± 17.23		
Bachelor’s	46.75 ± 18.96			45.02 ± 18.36			55.81 ± 17.99		
Master’s or above	45.83 ± 18.32			42.29 ± 14.79			56.04 ± 20.52		
Gender		−0.456	0.648		−1.202	0.229		0.879	0.380
Male	44.85 ± 18.20			42.36 ± 17.80			57.56 ± 16.52		
Female	45.65 ± 18.85			44.37 ± 17.90			56.10 ± 18.00		
Region		62.660	0.001**		31.598	0.001**		23.232	0.001**
Western	38.59 ± 14.53			39.44 ± 15.88			60.60 ± 15.80		
Central	47.14 ± 19.92			44.90 ± 18.06			54.86 ± 18.08		
Eastern	53.30 ± 19.07			49.76 ± 18.61			52.02 ± 18.92		
Age		1.666	0.173		1.446	0.228		2.834	0.037*
≤25	46.05 ± 18.65			43.48 ± 17.35			55.93 ± 17.47		
26 ~ 35	46.15 ± 18.97			44.81 ± 18.13			55.41 ± 17.73		
36 ~ 45	44.59 ± 19.04			44.35 ± 18.30			57.51 ± 18.74		
≥46	41.40 ± 16.58			40.53 ± 16.70			61.32 ± 16.97		
Years of work experience		1.224	0.299		1.145	0.334		2.172	0.070
1 ~ 5	46.46 ± 18.79			44.66 ± 18.41			55.47 ± 17.65		
6 ~ 10	45.44 ± 18.91			43.18 ± 17.10			55.84 ± 17.69		
11 ~ 15	46.00 ± 19.49			45.43 ± 18.21			55.96 ± 18.23		
16 ~ 20	44.96 ± 18.71			45.54 ± 18.27			56.33 ± 18.18		
≥21	42.29 ± 17.19			41.87 ± 17.19			60.72 ± 17.72		
Position		1.011	0.364		1.431	0.239		0.132	0.876
Staff nurse	45.40 ± 18.77			44.02 ± 17.90			56.34 ± 17.83		
Preceptor	48.97 ± 19.51			47.58 ± 18.21			55.76 ± 18.30		
Head nurse or above	44.09 ± 16.52			40.76 ± 16.13			54.55 ± 17.20		
Professional title		0.660	0.577		1.996	0.113		0.278	0.841
Nurse	44.35 ± 18.10			42.10 ± 16.86			56.69 ± 17.11		
Nurse practitioner	46.07 ± 18.79			45.23 ± 18.38			55.80 ± 17.75		
Charge nurse	46.05 ± 19.54			44.41 ± 18.05			56.50 ± 18.77		
Associate chief nurse or above	44.13 ± 17.91			43.73 ± 17.20			58.13 ± 18.13		
Employment type		−7.071	0.001**		−3.595	0.001**		5.367	0.001**
Permanent (In-staff)	40.07 ± 16.19			41.34 ± 16.82			60.44 ± 16.90		
Non-permanent (contract)	47.96 ± 19.32			45.36 ± 18.21			54.44 ± 17.93		
Marital status		0.241	0.809		−0.254	0.800		−0.112	0.911
Single	45.71 ± 18.74			43.97 ± 17.86			56.20 ± 17.60		
Married	45.44 ± 18.80			44.25 ± 17.93			56.32 ± 18.01		
Childbirth status		−0.083	0.934		0.393	0.694		−0.473	0.636
No children	45.51 ± 18.65			44.35 ± 17.83			56.01 ± 17.57		
Has children	45.60 ± 18.90			43.93 ± 17.96			56.52 ± 18.09		
Monthly income (10,000 RMB)		7.447	0.001**		1.121	0.339		6.154	0.001**
≤0.5	49.83 ± 20.51			45.41 ± 17.86			52.25 ± 18.14		
0.6 ~ 1.0	46.22 ± 18.88			44.64 ± 18.27			55.72 ± 18.18		
1.1 ~ 1.5	46.36 ± 18.62			43.87 ± 17.39			55.75 ± 17.58		
≥1.6	40.07 ± 16.50			42.12 ± 17.25			60.91 ± 15.99		
Daily surgical caseload (cases)		4.213	0.006**		2.925	0.033*		2.160	0.091
0 ~ 2	46.32 ± 18.85			46.08 ± 18.27			54.89 ± 18.72		
3 ~ 4	47.03 ± 19.01			44.85 ± 18.13			55.49 ± 17.85		
5 ~ 6	43.61 ± 18.26			42.96 ± 17.28			57.60 ± 17.51		
≥7	41.18 ± 17.69			40.32 ± 17.04			59.24 ± 16.90		
Daily overtime (h)		2.252	0.081		1.412	0.238		0.636	0.592
No overtime	49.80 ± 19.22			47.41 ± 17.38			55.22 ± 18.09		
1 ~ 2	45.85 ± 18.54			44.34 ± 18.03			55.76 ± 18.26		
3 ~ 4	44.57 ± 18.99			43.10 ± 17.63			57.07 ± 17.20		
≥5	42.85 ± 18.30			44.18 ± 18.77			57.70 ± 17.39		
Work pattern		−1.681	0.093		−1.294	0.196		2.159	0.031*
Fixed day shift	44.56 ± 18.47			43.40 ± 17.63			57.48 ± 17.40		
Rotating shift	46.44 ± 19.00			44.79 ± 18.12			55.19 ± 18.15		
Shift rotation pattern		5.550	0.004**		4.606	0.010**		5.094	0.006**
Regular	48.03 ± 19.08			46.16 ± 18.23			53.72 ± 18.51		
Irregular	42.74 ± 18.33			40.74 ± 16.64			58.95 ± 16.94		
Flexible	41.32 ± 18.14			42.43 ± 19.20			59.10 ± 16.09		
Monthly night shifts		1.937	0.122		1.328	0.264		2.682	0.046*
No night shifts	44.86 ± 18.33			43.75 ± 17.58			57.28 ± 17.39		
1 ~ 2	48.32 ± 19.19			46.40 ± 19.25			53.15 ± 18.22		
3 ~ 4	45.46 ± 19.47			43.41 ± 17.46			56.50 ± 17.96		
≥5	43.78 ± 17.80			43.69 ± 17.90			56.09 ± 19.00		
Interval between night shifts (d)		4.809	0.009**		1.322	0.268		3.087	0.046*
≤5	42.21 ± 18.31			42.21 ± 17.37			58.53 ± 18.62		
6 ~ 12	45.41 ± 19.21			44.20 ± 18.08			55.82 ± 18.05		
≥13	50.05 ± 19.05			46.31 ± 18.82			52.42 ± 18.20		
Daily sleep duration (h)		5.260	0.001**		4.386	0.004**		0.820	0.483
≤5	41.60 ± 17.74			41.05 ± 17.09			57.67 ± 16.84		
5.1 ~ 6	43.88 ± 18.26			42.70 ± 17.39			56.70 ± 17.82		
6.1 ~ 7	46.37 ± 18.84			44.71 ± 17.98			56.24 ± 17.89		
≥7	50.23 ± 19.77			48.48 ± 18.92			54.19 ± 18.24		
Competency level		5.714	0.001**		3.319	0.006**		1.562	0.168
N0	47.60 ± 19.95			44.65 ± 17.55			55.47 ± 17.24		
N1	47.99 ± 18.06			45.52 ± 18.63			54.58 ± 17.61		
N2	42.15 ± 17.87			41.63 ± 17.30			57.88 ± 17.32		
N3	46.49 ± 19.34			45.61 ± 17.77			56.30 ± 18.83		
N4	49.70 ± 20.07			48.75 ± 18.96			53.87 ± 18.94		

*p < 0.05; **p < 0.01; post-hoc pairwise comparisons for multiple groups used the LSD method.

### Correlation analysis among acute fatigue, chronic fatigue, inter-shift recovery, job content, and perceived stress

The results of Pearson correlation analysis are shown in Table 5. At the level of work characteristics and support resources, job demand was weakly negatively correlated with job control (*r* = −0.089) and social support (*r* = −0.098), while job control was positively correlated with social support (*r* = 0.265). At the level of stress and fatigue, both job demand and perceived stress were significantly positively correlated with acute fatigue and chronic fatigue; among these, job demand had the strongest correlation with acute fatigue (*r* = 0.503), and perceived stress had the strongest correlation with acute fatigue (*r* = 0.557). Job control and social support were significantly negatively correlated with acute fatigue and chronic fatigue, and significantly positively correlated with inter-shift recovery. Acute fatigue and chronic fatigue were highly positively correlated (*r* = 0.550), suggesting that fatigue has cumulative and persistent characteristics. The absolute values of correlation coefficients among all variables were less than 0.8; combined with the result that all VIF values in hierarchical regression were <2, this indicates no serious multicollinearity.

**Table 5:** Correlation analysis among acute fatigue, chronic fatigue, inter-shift recovery, job content, and perceived stress (*n* = 1,122).

Variable	Job demand	Job control	Social support	Perceived stress	Acute fatigue	Chronic fatigue	Inter-shift recovery
Job demand	1						
Job control	−0.089**	1					
Social support	−0.098**	0.265**	1				
Perceived stress	0.350**	−0.154**	−0.316**	1			
Acute fatigue	0.503**	−0.237**	−0.380**	0.557**	1		
Chronic fatigue	0.265**	−0.313**	−0.430**	0.452**	0.550**	1	
Inter-shift recovery	−0.295**	0.312**	0.481**	−0.420**	−0.569**	−0.566**	1

**p < 0.01.

### Hierarchical regression analysis

This study used hierarchical multiple linear regression models, entering control variables and core independent variables layer by layer, to progressively test the incremental predictive effects of job demand, job control, and social support on acute fatigue, chronic fatigue, and inter-shift recovery, while strictly testing for multicollinearity through tolerance and VIF values. The VIF values of all model variables were less than 2 and tolerance values were greater than 0.6, indicating no multicollinearity; the model regression results were stable and valid, as shown in Tables 6, 7, 8.

**Table 6:** Hierarchical regression analysis for acute fatigue (AF).

Model	Variable	B	Std. Error	Beta	t	Sig.	Tolerance	VIF	R^2^	F
1	(Constant)	30.939	7.734		4.000	<0.001			0.166	12.847***
	Education	4.403	1.649	0.107	2.670	0.008	0.893	1.119		
	Region	7.293	1.206	0.289	6.045	<0.001	0.629	1.590		
	Employment type	2.153	2.104	0.048	1.024	0.306	0.657	1.523		
	Monthly income	−0.449	1.107	−0.019	−0.406	0.685	0.637	1.570		
	Daily surgical caseload	−1.378	0.967	−0.056	−1.424	0.155	0.928	1.078		
	Shift rotation pattern	−3.544	1.170	−0.117	−3.030	0.003	0.962	1.040		
	Daily sleep duration	0.782	1.023	0.031	0.765	0.445	0.882	1.134		
	Competency level	1.500	0.811	0.078	1.850	0.065	0.799	1.252		
2	(Constant)	−30.677	8.259		−3.715	<0.001			0.357	32.225***
	Education	2.878	1.454	0.070	1.980	0.048	0.888	1.126		
	Region	5.426	1.070	0.215	5.072	<0.001	0.618	1.618		
	Employment type	2.074	1.849	0.046	1.122	0.262	0.657	1.523		
	Monthly income	0.122	0.973	0.005	0.126	0.900	0.636	1.573		
	Daily surgical caseload	−1.244	0.850	−0.051	−1.463	0.144	0.928	1.078		
	Shift rotation pattern	−3.478	1.028	−0.115	−3.383	0.001	0.962	1.040		
	Daily sleep duration	1.350	0.900	0.053	1.500	0.134	0.880	1.137		
	Competency level	0.536	0.716	0.028	0.748	0.454	0.791	1.265		
	Total job demand score	1.775	0.135	0.451	13.136	<0.001	0.938	1.066		
3	(Constant)	−18.440	8.536		−2.160	0.031			0.380	32.280***
	Education	2.930	1.429	0.071	2.051	0.041	0.888	1.127		
	Region	5.129	1.053	0.203	4.869	<0.001	0.616	1.624		
	Employment type	2.116	1.817	0.047	1.165	0.245	0.657	1.523		
	Monthly income	0.027	0.957	0.001	0.028	0.978	0.635	1.574		
	Daily surgical caseload	−1.307	0.836	−0.053	−1.564	0.118	0.928	1.078		
	Shift rotation pattern	−3.148	1.013	−0.104	−3.108	0.002	0.957	1.045		
	Daily sleep duration	1.816	0.890	0.072	2.041	0.042	0.868	1.152		
	Competency level	0.290	0.706	0.015	0.411	0.681	0.786	1.272		
	Total job demand score	1.739	0.133	0.442	13.079	<0.001	0.935	1.070		
	Total job control score	−0.165	0.036	−0.155	−4.634	<0.001	0.958	1.044		
4	(Constant)	−2.045	8.169		−0.250	0.802			0.460	41.030***
	Education	3.815	1.338	0.093	2.851	0.005	0.883	1.132		
	Region	4.931	0.984	0.195	5.010	<0.001	0.615	1.625		
	Employment type	1.214	1.700	0.027	0.714	0.476	0.654	1.528		
	Monthly income	0.129	0.894	0.006	0.144	0.885	0.635	1.574		
	Daily surgical caseload	−1.203	0.781	−0.049	−1.541	0.124	0.927	1.078		
	Shift rotation pattern	−2.490	0.949	−0.082	−2.623	0.009	0.952	1.051		
	Daily sleep duration	1.685	0.832	0.066	2.026	0.043	0.868	1.152		
	Competency level	0.316	0.660	0.016	0.479	0.632	0.786	1.272		
	Total job demand score	1.636	0.125	0.416	13.116	<0.001	0.927	1.078		
	Total job control score	−0.081	0.034	−0.076	−2.351	0.019	0.892	1.121		
	Total social support score	−0.856	0.093	−0.299	−9.248	<0.001	0.894	1.119		

**p < 0.01; ***p < 0.001.

**Table 7:** Hierarchical regression analysis for chronic fatigue (CF).

Model	Variable	B	Std. Error	Beta	t	Sig.	Tolerance	VIF	R^2^	F
1	(Constant)	32.011	6.075		5.269	<0.001			0.080	7.291***
	Education	2.069	1.611	0.053	1.285	0.199	0.935	1.070		
	Region	5.071	1.140	0.210	4.449	<0.001	0.703	1.422		
	Employment Type	−1.022	2.053	−0.024	−0.498	0.619	0.688	1.453		
	Daily Surgical Caseload	−1.344	0.962	−0.057	−1.396	0.163	0.936	1.068		
	Shift Rotation Pattern	−2.482	1.162	−0.086	−2.137	0.033	0.974	1.027		
	Daily Sleep Duration	1.104	1.016	0.046	1.087	0.277	0.892	1.121		
	Competency Level	1.579	0.779	0.087	2.027	0.043	0.864	1.157		
2	(Constant)	5.528	7.562		0.731	0.465			0.128	10.678***
	Education	1.337	1.575	0.034	0.849	0.396	0.928	1.077		
	Region	4.167	1.123	0.173	3.713	<0.001	0.689	1.451		
	Employment Type	−1.220	2.001	−0.028	−0.610	0.542	0.688	1.454		
	Daily Surgical Caseload	−1.334	0.938	−0.057	−1.422	0.155	0.936	1.068		
	Shift Rotation Pattern	−2.483	1.132	−0.086	−2.193	0.029	0.974	1.027		
	Daily Sleep Duration	1.311	0.991	0.054	1.323	0.186	0.891	1.123		
	Competency Level	1.203	0.762	0.066	1.578	0.115	0.857	1.166		
	Total Job Demand Score	0.837	0.149	0.223	5.631	<0.001	0.950	1.052		
3	(Constant)	26.911	7.849		3.429	0.001			0.198	15.988***
	Education	1.314	1.512	0.033	0.869	0.385	0.928	1.077		
	Region	3.789	1.078	0.157	3.513	<0.001	0.687	1.455		
	Employment Type	−1.128	1.920	−0.026	−0.587	0.557	0.688	1.454		
	Daily Surgical Caseload	−1.462	0.900	−0.062	−1.624	0.105	0.936	1.069		
	Shift Rotation Pattern	−1.916	1.089	−0.066	−1.759	0.079	0.969	1.032		
	Daily Sleep Duration	2.065	0.957	0.085	2.159	0.031	0.880	1.137		
	Competency Level	0.785	0.734	0.043	1.070	0.285	0.852	1.174		
	Total Job Demand Score	0.772	0.143	0.206	5.401	<0.001	0.946	1.057		
	Total Job Control Score	−0.274	0.038	−0.270	−7.150	<0.001	0.963	1.038		
4	(Constant)	46.686	7.606		6.138	<0.001			0.305	25.514***
	Education	2.265	1.412	0.058	1.604	0.109	0.924	1.083		
	Region	3.591	1.005	0.149	3.573	<0.001	0.687	1.455		
	Employment Type	−2.203	1.793	−0.051	−1.229	0.220	0.685	1.459		
	Daily Surgical Caseload	−1.381	0.839	−0.059	−1.646	0.100	0.936	1.069		
	Shift Rotation Pattern	−1.206	1.018	−0.042	−1.185	0.236	0.964	1.038		
	Daily Sleep Duration	1.878	0.892	0.078	2.107	0.036	0.879	1.137		
	Competency Level	0.858	0.684	0.047	1.255	0.210	0.852	1.174		
	Total Job Demand Score	0.652	0.134	0.174	4.869	<0.001	0.938	1.066		
	Total Job Control Score	−0.181	0.037	−0.178	−4.878	<0.001	0.895	1.118		
	Total Social Support Score	−0.943	0.100	−0.345	−9.455	<0.001	0.896	1.116		

**p < 0.01; ***p < 0.001.

**Table 8:** Hierarchical regression analysis for inter-shift recovery (IR).

Model	Variable	*B*	Std. Error	Beta	*t*	Sig.	Tolerance	VIF	*R* ^2^	*F*
1	(Constant)	68.385	9.760		7.006	<0.001			0.070	5.903***
	Region	−4.763	1.253	−0.192	−3.801	<0.001	0.663	1.508		
	Age	−0.742	1.440	−0.025	−0.515	0.607	0.740	1.352		
	Employment type	−1.318	2.372	−0.030	−0.555	0.579	0.584	1.711		
	Monthly income	0.279	1.131	0.012	0.247	0.805	0.668	1.498		
	Shift rotation pattern	3.627	1.218	0.125	2.979	0.003	0.963	1.039		
	Monthly night shifts	0.328	1.479	0.012	0.222	0.824	0.580	1.725		
	Interval between night shifts	−3.064	1.637	−0.102	−1.872	0.062	0.570	1.756		
2	(Constant)	101.021	10.574		9.554	<0.001			0.141	11.212***
	Region	−3.507	1.220	−0.142	−2.875	0.004	0.648	1.544		
	Age	−0.102	1.388	−0.003	−0.073	0.942	0.736	1.358		
	Employment type	−1.364	2.282	−0.031	−0.598	0.550	0.584	1.711		
	Monthly income	0.078	1.088	0.003	0.071	0.943	0.667	1.499		
	Shift rotation pattern	3.594	1.171	0.124	3.068	0.002	0.963	1.039		
	Monthly night shifts	0.204	1.423	0.007	0.143	0.886	0.580	1.725		
	Interval between night shifts	−2.005	1.582	−0.067	−1.267	0.206	0.564	1.773		
	Total job demand score	−1.037	0.155	−0.274	−6.711	<0.001	0.943	1.061		
3	(Constant)	79.733	10.636		7.497	<0.001			0.208	15.910***
	Region	−3.083	1.174	−0.125	−2.625	0.009	0.646	1.549		
	Age	−0.058	1.334	−0.002	−0.043	0.965	0.736	1.358		
	Employment type	−2.013	2.195	−0.046	−0.917	0.359	0.583	1.714		
	Monthly income	0.332	1.047	0.015	0.317	0.751	0.666	1.501		
	Shift rotation pattern	3.129	1.128	0.108	2.774	0.006	0.959	1.043		
	Monthly night shifts	0.127	1.368	0.005	0.093	0.926	0.580	1.725		
	Interval between night shifts	−1.450	1.523	−0.048	−0.952	0.341	0.562	1.778		
	Total job demand score	−0.997	0.149	−0.263	−6.706	<0.001	0.941	1.062		
	Total job control score	0.267	0.039	0.261	6.789	<0.001	0.980	1.020		
4	(Constant)	55.987	9.863		5.677	<0.001			0.353	29.636***
	Region	−2.398	1.064	−0.097	−2.253	0.025	0.644	1.554		
	Age	−0.717	1.209	−0.024	−0.593	0.553	0.734	1.362		
	Employment type	−1.415	1.987	−0.032	−0.712	0.477	0.583	1.716		
	Monthly income	0.336	0.947	0.015	0.355	0.723	0.666	1.501		
	Shift rotation pattern	2.295	1.023	0.079	2.243	0.025	0.954	1.049		
	Monthly night shifts	0.033	1.238	0.001	0.026	0.979	0.580	1.725		
	Interval between night shifts	−1.330	1.378	−0.044	−0.965	0.335	0.562	1.779		
	Total job demand score	−0.861	0.135	−0.228	−6.374	<0.001	0.933	1.071		
	Total job control score	0.163	0.037	0.160	4.449	<0.001	0.917	1.091		
	Total social support score	1.103	0.100	0.400	11.023	<0.001	0.903	1.107		

**p < 0.01; ***p < 0.001.

With acute fatigue as the dependent variable (Table 6): Model 1 included only demographic variables, and the overall model was significant (*F* = 12.847, *p* < 0.001), with an explanatory power *R*^2^ of 0.166. Region (*β* = 0.289, *p* < 0.001) and education (*β* = 0.107, *p* = 0.008) positively predicted acute fatigue, while shift rotation pattern (*β* = −0.117, *p* = 0.003) negatively predicted acute fatigue. After sequentially entering job demand, job control, and social support, Model 4 achieved the optimal overall explanatory power (*R*^2^ = 0.460, *F* = 41.030, *p* < 0.001); job demand (*β* = 0.416, *p* < 0.001) was significantly positively correlated with acute fatigue, while job control (*β* = −0.076, *p* = 0.019) and social support (*β* = −0.299, *p* < 0.001) were significantly negatively correlated; region, education, shift rotation pattern, and sleep duration remained independent correlates.

With chronic fatigue as the dependent variable (Table 7): Model 1 had *R*^2^ = 0.080 (*F* = 7.291, *p* < 0.001), with region (*β* = 0.210, *p* < 0.001) and competency level (*β* = 0.087, *p* = 0.043) positively predicting chronic fatigue; Model 4 had *R*^2^ increased to 0.305 (*F* = 25.514, *p* < 0.001), with job demand (*β* = 0.174, *p* < 0.001) positively, and job control (*β* = −0.178, *p* < 0.001) and social support (*β* = −0.345, *p* < 0.001) negatively predicting chronic fatigue; region and sleep duration remained significant.

With inter-shift recovery as the dependent variable (Table 8): Model 1 had *R*^2^ = 0.070 (*F* = 5.903, *p* < 0.001), with region (*β* = −0.192, *p* < 0.001) negatively and shift rotation regularity (*β* = 0.125, *p* = 0.003) positively predicting inter-shift recovery; Model 4 had R^2^ increased to 0.353 (*F* = 29.636, *p* < 0.001), with job demand (*β* = −0.228, *p* < 0.001) negatively, and job control (*β* = 0.160, *p* < 0.001) and social support (*β* = 0.400, *p* < 0.001) positively predicting inter-shift recovery, among which social support had the strongest predictive effect.

### Structural equation model of job demand, job control, social support, perceived stress, and occupational fatigue

This study constructed a structural equation model to examine the mechanisms among job demand (JD), job control (JC), social support (SS), perceived stress (PS), acute fatigue (AF), chronic fatigue (CF), and inter-shift recovery (IR), and tested the mediating effect of perceived stress through the Bootstrap method. The overall model fit results showed that CMIN/DF = 2.562, below the strict fit standard of 3, indicating good overall structural fit; RMSEA = 0.048 and RMR = 0.051, with residual errors within a controllable range; IFI = 0.949, TLI = 0.945, and CFI = 0.949, all incremental fit indices significantly above the 0.9 standard, while GFI and AGFI were close to the 0.9 critical value. Comprehensive judgment indicated a high match between the structural model and the sample data, with a stable and reliable model structure suitable for path mechanism testing, as shown in Figure 3.

**Figure 3:**
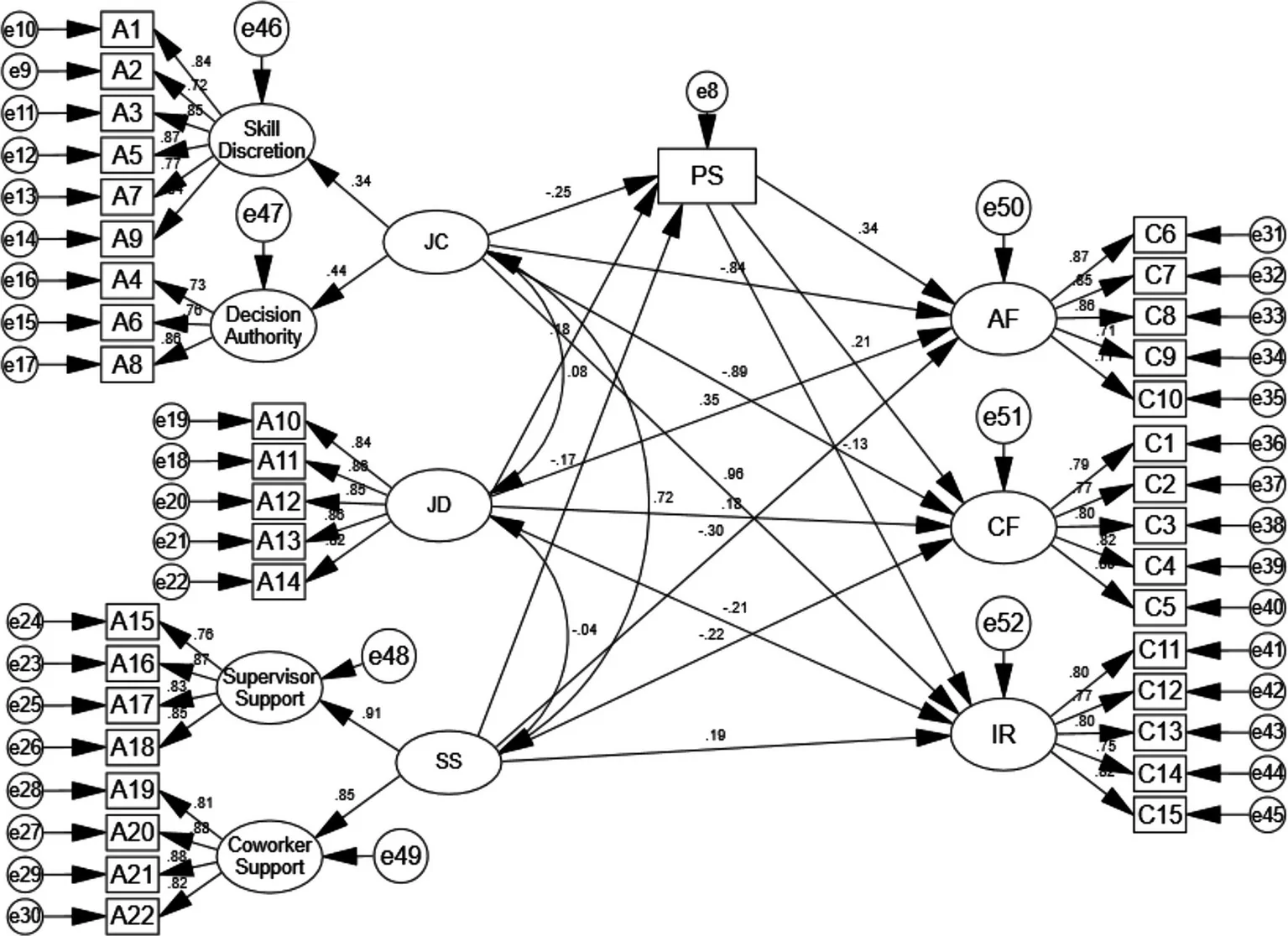
Structural equation model of operating room nurses’ occupational fatigue and its influencing factors.

The path coefficients (Table 1) showed that: on the stress formation pathway, job demand positively predicted perceived stress, while job control and social support negatively predicted perceived stress, supporting hypothesis H5. On the outcome pathway: perceived stress positively predicted acute fatigue and chronic fatigue and negatively predicted inter-shift recovery, supporting hypothesis H4; job demand positively predicted acute fatigue and chronic fatigue and negatively predicted inter-shift recovery, supporting hypothesis H1; job control negatively predicted acute fatigue and chronic fatigue and positively predicted inter-shift recovery, supporting hypothesis H2; social support negatively predicted acute fatigue and chronic fatigue and positively predicted inter-shift recovery, supporting hypothesis H3.

The results of the mediating effect analysis are shown in Table 9. The bias-corrected Bootstrap method (5,000 resamples) showed that: perceived stress played a partial mediating role between job demand and acute fatigue, chronic fatigue, and inter-shift recovery, supporting hypothesis H6; perceived stress played a partial mediating role between job control and acute fatigue, chronic fatigue, and inter-shift recovery, supporting hypothesis H7; perceived stress played a partial mediating role between social support and acute fatigue, chronic fatigue, and inter-shift recovery, supporting hypothesis H8. The indirect effects were small in magnitude (absolute values 0.023–0.084), and the proportions of the total effects mediated by perceived stress ranged from 3.41% (job control → inter-shift recovery) to 17.35% (job demand → chronic fatigue), indicating that, although statistically significant, the mediating effects were modest relative to the direct effects (Table 9).

**Table 9:** Mediating effect analysis of perceived stress.

Antecedent variable	Mediating pathway	Direct effect	Indirect effect	95% CI (indirect)	Total effect	Hypothesis result	Proportion mediated (%)
Job demand	Job demand → perceived stress → acute fatigue	0.349	0.061	0.042 ~ 0.088	0.410	H6a supported	14.88
Job demand	Job demand → perceived stress → chronic fatigue	0.181	0.038	0.021 ~ 0.069	0.219	H6b supported	17.35
Job demand	Job demand → perceived stress → inter-shift recovery	−0.213	−0.024	−0.049 ~ −0.005	−0.237	H6c supported	10.13
Job control	Job control → perceived stress → acute fatigue	−0.844	−0.084	−0.133 ~ −0.031	−0.928	H7a supported	9.05
Job control	Job control → perceived stress → chronic fatigue	−0.888	−0.053	−0.156 ~ −0.040	−0.941	H7b supported	5.63
Job control	Job control → perceived stress → inter-shift recovery	0.963	0.034	0.021 ~ 0.049	0.997	H7c supported	3.41
Social support	Social support → perceived stress → acute fatigue	−0.301	−0.057	−0.136 ~ −0.017	−0.358	H8a supported	15.92
Social support	Social support → perceived stress → chronic fatigue	−0.219	−0.035	−0.107 ~ −0.002	−0.254	H8b supported	13.78
Social support	Social support → perceived stress → inter-shift recovery	0.194	0.023	0.001 ~ 0.084	0.217	H8c supported	10.60

Standardized estimates are reported. Proportion mediated = indirect effect / total effect × 100% (absolute values).

## Discussion

### Current status of occupational fatigue, perceived stress, and job content among operating room nurses

In this study, acute fatigue and chronic fatigue among 1,122 operating room nurses from 9 provinces, municipalities, and autonomous regions were at moderate to severe levels, and inter-shift recovery still had considerable room for improvement. Compared with foreign studies, the acute fatigue scores of operating room nurses in China were slightly lower than those of American anesthesia nurses and pediatric nurses (31, 32), but chronic fatigue was significantly higher, inter-shift recovery scores were lower than those of American anesthesia nurses, and the prevalence of moderate to severe acute and chronic fatigue was higher than that of American anesthesia nurses. Compared with other domestic populations, the fatigue scores and prevalence of nurses in this study were also higher than those of nurses in general hospitals in Chengdu, and inter-shift recovery was slightly lower than that of new nurses in Taiwan, China (26, 33). Operating room work is characterized by high intensity, prolonged standing, frequent emergencies, high risk, and high mental tension; nurses are in a state of dual physical and mental consumption for a long time, and the problems of fatigue accumulation and insufficient recovery deserve continuous attention.

The perceived stress score in this study was 21.01 ± 5.24, at a moderate to high level, indicating that operating room nurses generally perceive high levels of stress. In this study, the scores of job demand and social support among operating room nurses were slightly higher than those of Japanese nurses, while the job control score was lower than that of Japanese nurses; compared with domestic clinical nurses, all three scores of operating room nurses in this study were higher (34–36). The results present a work characteristic of “high demand, moderate control, and moderate support,” which is consistent with the high-stress job characteristic of “high job demand—low decision latitude” in the JDCS model (12). This also indicates that the operating room is in the high-stress work situation defined by the JDCS model, which explains why occupational fatigue is at a relatively high level.

### Differences in fatigue among populations with different characteristics

In terms of population differences, regional differences were most prominent across all three outcomes: nurses in the eastern region had the highest fatigue scores and the worst inter-shift recovery, while the opposite was true for the western region. This may be related to the concentration of medical resources, large surgical volumes, and fast work pace in the eastern region, and may also reflect differences in staffing and management models among hospitals in different regions. Hierarchical regression further confirmed that region was an independent correlate of acute and chronic fatigue. Regarding employment type, non-permanent nurses had higher fatigue and worse recovery, reflecting differences in job stability and resource security; the job security brought by permanent status may alleviate fatigue by reducing occupational insecurity. In hierarchical regression, employment type was no longer significant after social support was entered. Because this study is cross-sectional, this attenuation reflects shared variance between employment type and social support rather than evidence of causal mediation; whether employment status operates through social support should be tested in longitudinal designs. Regarding monthly income, the low-income group had the highest acute fatigue and the worst recovery, while the high-income group showed the opposite, suggesting that economic security may be a protective factor for alleviating fatigue; however, it was not significant in hierarchical regression, possibly because its association with fatigue overlaps with core variables such as job demand and job control. Regarding education, nurses with a bachelor’s degree or above had the highest acute fatigue scores, possibly because highly educated nurses bear more technical responsibilities and assessment pressure. Education remained significant after core variables were entered in hierarchical regression, suggesting that education-related fatigue risk cannot be fully explained by work characteristics. Regarding competency level, N2-level nurses had the lowest fatigue and N4-level nurses had the highest, possibly because N4-level nurses bear more management, teaching, and complex surgical coordination tasks, similar to previous research results (37). The reason may be that the post setting in Chinese operating rooms is still in the exploratory stage; the imperfect post-setting system and the shortage of human resources lead high-competency operating room nurses to bear the dual load of both mentoring low-competency clinical nurses and undertaking clinical work, doubling their stress (38–40). However, competency level was not significant after core variables were entered in hierarchical regression, indicating that its association with fatigue largely overlaps with the work characteristics included in the model; again, this attenuation should not be interpreted as evidence of causal mediation.

Night-shift-related characteristics showed a complex nonlinear pattern. Regarding shift rotation pattern, nurses with regular rotating shifts had the highest fatigue scores, while those with irregular and flexible rotating shifts had lower scores; those with ≥5 monthly night shifts had significantly higher chronic fatigue than those without night shifts; those with ≥13-day intervals between night shifts had the highest acute fatigue, significantly higher than the ≤5-day group. These three findings point to the same core issue: night-shift-related fatigue depends on the interaction between “cumulative exposure” and “circadian adaptation status,” rather than whether the scheduling pattern itself is “regular.” “Regular” in this study meant fixed night-shift assignments, with the highest actual night-shift frequency, hence the heaviest fatigue accumulation; when the interval between night shifts was too long, the circadian rhythm fully recovered to the day-shift mode, and re-engagement required a drastic “restart” stress response, with greater physiological cost. Hierarchical regression showed that shift rotation pattern remained significant after core variables were entered, while monthly number of night shifts and interval between night shifts were no longer significant, suggesting that the associations of night-shift frequency and interval with fatigue largely overlap with (share variance with) the psychosocial variables, whereas shift rotation pattern remained associated with fatigue independently of these factors. In this cross-sectional design, such attenuation should be interpreted as shared variance rather than as evidence of causal mediation. Regarding daily surgical caseload, nurses with 0–2 cases/day had higher acute fatigue scores than those with ≥7 cases/day. A possible explanation is that hospitals with large surgical volumes have more mature staffing and process management, with high workload but structural support; whereas those with low surgical volumes may undertake more fragmented work due to redundant staffing, and “passive standby” under low workload itself constitutes psychological consumption. Daily surgical caseload was no longer significant after core variables were entered in hierarchical regression, suggesting that its association with fatigue largely overlaps with the core psychosocial variables rather than reflecting a distinct causal pathway. The relationship between daily sleep duration and fatigue is noteworthy: nurses with ≥7 h of average daily sleep had the highest fatigue scores, while those with ≤5 h had the lowest, which isclearly inconsistent with conventional understanding. This may be related to increased sleep demand caused by fatigue, prolonged bed rest due to poor sleep quality but insufficient recovery, and the bidirectional causal relationship between fatigue and sleep. In hierarchical regression, sleep duration remained independently associated with acute fatigue and chronic fatigue after job demand, control, and support were entered, suggesting that the association of sleep with fatigue is not fully accounted for by the work characteristics included in the model; future studies should use objective sleep monitoring for further verification.

### Direct effects of JDCS dimensions on fatigue and inter-shift recovery outcomes

Through the structural equation model, this study found that the dimensions of the JDCS model had differentiated direct effects on acute fatigue, chronic fatigue, and inter-shift recovery outcomes: job demand was the main risk factor, while job control and social support were important protective resources.

Job demand had a significant positive predictive effect on acute fatigue and chronic fatigue, and a significant negative predictive effect on inter-shift recovery. Among these, the effect size on acute fatigue was the largest, suggesting that immediate workload overload is the main trigger of acute fatigue. This result is consistent with the core proposition of the JDCS model that high job demand is the main threat to occupational health, and is consistent with previous studies (41, 42). It is worth noting that the effect size of job demand on chronic fatigue was about half of that on acute fatigue, indicating that long-term cumulative fatigue not only stems from immediate workload but may also be associated with fatigue indirectly through pathways involving perceived stress. Hierarchical regression also confirmed the independent predictive effect of job demand on acute fatigue and chronic fatigue, with the largest effect size on acute fatigue, further supporting this conclusion.

Both job control and social support had significant protective effects on all three outcomes: negatively predicting acute fatigue and chronic fatigue, and positively predicting inter-shift recovery, consistent with domestic and international research results (43–45). Standardized coefficients showed that the effect sizes of job control on chronic fatigue and inter-shift recovery were larger than those on acute fatigue, indicating that control resources such as work autonomy and decision-making participation play a more important role in alleviating long-term cumulative fatigue and promoting recovery. When nurses have a higher sense of control over work content, pace, and decision-making processes, they can more effectively allocate energy and cope with stress, thereby alleviating fatigue and promoting recovery. Hierarchical regression also confirmed that, after controlling for other variables, job control still independently and negatively predicted acute fatigue and chronic fatigue. The effect size of social support on acute fatigue was larger than that on chronic fatigue, suggesting that emotional support, collaborative assistance, and professional guidance from superiors and colleagues have a more direct effect in alleviating immediate fatigue. Social support constitutes an important “buffering system” for nurses coping with high-pressure environments. Hierarchical regression showed that social support was one of the strongest predictors of acute fatigue, chronic fatigue, and inter-shift recovery, indicating that social support plays an irreplaceable role in alleviating occupational fatigue. Nursing managers should strive to build a supportive organizational culture, strengthen head nurse leadership training, and promote teamwork.

### Mediating role of perceived stress

In the stress formation pathway, job control had a significant negative effect on perceived stress, social support significantly negatively predicted perceived stress, and job demand significantly positively increased perceived stress, indicating that high workload exacerbates employee stress, while work autonomy and external support resources can effectively alleviate perceived stress. In the fatigue and recovery outcome pathway, perceived stress significantly positively predicted acute fatigue and chronic fatigue and significantly negatively affected inter-shift recovery, consistent with perceived stress being an important correlate of occupational fatigue and weakened energy recovery. Meanwhile, job control had a significant negative effect on acute fatigue and chronic fatigue and a significant positive effect on inter-shift recovery; job demand significantly exacerbated acute and chronic fatigue and inhibited inter-shift recovery; social support effectively reduced both types of fatigue and promoted inter-shift energy recovery. The direct associations among variables are consistent with the expectations of the stress-resource theory.

The data showed that perceived stress had statistically significant partial mediating effects between the three antecedent variables (job demand, job control, and social support) and the outcome variables (acute fatigue, chronic fatigue, and inter-shift recovery); the 95% confidence intervals of all mediating pathways did not contain 0. However, the indirect effects were small in magnitude (0.023–0.084 in absolute value) relative to the direct effects, with proportions mediated ranging from 3.41 to 17.35%, indicating that the mediating effects, although statistically significant, were modest. Moreover, in this cross-sectional design these estimates should be interpreted as patterns of association consistent with partial mediation rather than as evidence of causal mediation. First, job demand not only directly and positively exacerbated acute fatigue and chronic fatigue and directly inhibited inter-shift energy recovery, but also exerted significant indirect effects by increasing employees’ perceived stress, with indirect effect values of 0.061, 0.038, and −0.024, respectively. These patterns are consistent with the view that excessive job task load is associated with higher stress appraisals, which in turn are associated with more severe short-term and long-term exhaustion and poorer inter-shift recovery, forming a negative chain pattern of “high job demand—high stress—high fatigue, low recovery.” Second, job control had both direct effects and mediating buffering effects on fatigue and recovery variables, with indirect effect values of −0.084, −0.053, and 0.034, respectively. Higher job control was associated with stronger work autonomy and sense of control, which were associated with lower perceived stress levels and, in turn, with less acute and chronic fatigue and better inter-shift recovery. These results are consistent with the view that job resources may alleviate occupational exhaustion and promote energy recovery partly by weakening perceived stress. Third, social support had a significant protective mediating mechanism, with indirect effect values of −0.057, −0.035, and 0.023, respectively; support resources from superiors and colleagues were associated with lower perceived stress, which in turn was associated with less acute and chronic fatigue and better inter-shift recovery.

### Implications for nursing management practice and research value

This study provides multi-target intervention ideas for nursing management. First, reduce burden and stress: through scientific human resource allocation, reasonable control of surgical scheduling and overtime hours, and establishment of a flexible nurse pool for deployment, reduce unreasonable job demands at the source and decrease the inducing factors of perceived stress. Second, empower and enable: managers should enhance nurses’ sense of job control and self-efficacy by authorizing participation in decision-making, enriching work content, and providing professional training. Meanwhile, stress management strategies can be implemented in coordination with empowerment measures to maximize the effect of alleviating acute fatigue. Third, strengthen support: strengthen head nurse support, build a team mutual-aid culture, and improve career development support; social support has the most significant promoting effect on inter-shift recovery and should be a key intervention direction for improving nurses’ recovery capacity. Fourth, stratified intervention: adopt differentiated strategies for different fatigue types: for acute fatigue, prioritize immediate measures (adjusting on-duty workload, ensuring rest time between shifts) combined with stress management interventions; for chronic fatigue and inter-shift recovery, long-term and systematic interventions at the organizational level, such as enhancing job control and strengthening social support, are needed. Meanwhile, high-risk groups should receive focused attention: nurses in the eastern region, non-permanent nurses, those with lower income and insufficient sleep, as well as N4 competency level and bachelor’s degree groups.

The theoretical contributions of this study are mainly reflected in the following three aspects. First, it expands the applicable boundaries of the JDCS model. This study is the first to apply the JDCS model to Chinese operating room nurses—a high-pressure, highly professional group—and simultaneously incorporates three outcome variables with different time dimensions (acute fatigue, chronic fatigue, and inter-shift recovery) into the model, providing evidence for the applicability of the model’s main effects in the operating room context. Second, it reveals the differentiated pattern of the mediating role of perceived stress. This study found that perceived stress played a statistically significant mediating role between job control and fatigue and inter-shift recovery outcomes, but with varying effect sizes—the proportion mediated for acute fatigue was larger than those for chronic fatigue and inter-shift recovery, consistent with a relatively larger contribution of the indirect “stress-buffering” pathway when alleviating acute fatigue, and a relatively larger contribution of the direct pathway when alleviating chronic fatigue and promoting recovery. This finding provides empirical evidence for understanding the multiple mechanisms of job control. Third, this study constructed and tested a comprehensive model including job demand, job control, social support, perceived stress, and three fatigue outcomes, providing a testable theoretical framework for future research.

## Limitations

This study has the following limitations. First, the cross-sectional design cannot infer causal relationships; although the hypothesized model is based on JDCS theory, the temporal order among variables cannot be determined, and reverse causality (e.g., fatigue may also exacerbate perceived stress) and bidirectional mutual influence are possible. Future longitudinal studies or cross-lagged designs are needed for verification. Second, the sample of this study was a multi-center convenience sample, with hospitals purposively selected and nurses sampled by convenience rather than probability sampling; therefore, the representativeness of the sample is limited, and the research conclusions cannot be generalized to all operating room nurses nationwide, being mainly applicable to the 9 hospitals participating in this study and nurse groups with similar backgrounds. Third, data collection relied on self-report, and all variables were collected at the same time point, which may introduce common method bias and social desirability effects. Future studies could be improved by combining objective behavioral observation, physiological indicator measurement, or mixed research paradigms. Fourth, the mediating variable was measured with the PSS-10, which assesses global, non-work-specific stress over the past month rather than work-specific job stress. Although the PSS-10 is a well-validated instrument and the mediator was labeled “perceived stress” throughout to reflect this operationalization, its content only partially overlaps with the work-related stress construct assumed in the JDCS model, and within the JDCS framework job demand and general perceived stress may overlap conceptually. A work-specific stress measure would have been preferable and should be used in future studies. Fifth, this study focused on the main effects of the JDCS model and did not test the buffering effects of job control and social support, which is one of the core assumptions of the JDCS model; future studies should use methods such as latent variable interaction structural equation models for testing. Sixth, the 1,122 nurses were nested within 9 hospitals, and the data may have clustering effects; this study did not use multilevel structural equation modeling. Future studies should include more hospitals to support multilevel modeling, thereby more accurately estimating the cross-level influence of the hospital environment on nurses’ occupational fatigue. Seventh, some results in univariate analysis (such as the positive association between sleep duration and fatigue) are inconsistent with conventional understanding, possibly related to reverse causality or unmeasured confounding in the cross-sectional design; future studies could use longitudinal or experimental designs, include objective physiological indicators and richer confounding variables, and combine multilevel modeling methods for improvement.

## Conclusion

Occupational fatigue among operating room nurses in 9 tertiary Grade-A hospitals is at a moderate level, and inter-shift recovery needs improvement. Under a unified measurement model, this study confirmed that job demand, job control, and social support are significantly associated with acute fatigue, chronic fatigue, and inter-shift recovery, and that perceived stress played a partial mediating role therein, although the indirect effects were modest in magnitude. The results provide preliminary evidence for the applicability of the additive main-effect pathway of the JDCS model among operating room nurses, and offer nursing managers multi-target intervention ideas such as reducing demand, enhancing control, strengthening support, and alleviating perceived stress.

## Data Availability

The anonymized dataset supporting the conclusions of this article is available from the corresponding author upon reasonable request. Requests to access these datasets should be directed to Xiaoning Wang, wxn20404@rjh.com.cn.

## References

[1] Zhao F Zhou XY Tian LL Gao Y Zhu MM Wang YY. Mediating effect of psychological capital between occupational fatigue and job burnout among operating room nurses. Chin J Mod Nurs. (2021) 27:4446–52. doi: 10.3760/cma.j.cn115682-20210305-00979

[2] Luo GQ. Correlation between work fatigue, pressure source and perceived control among operating room nurses in General Hospital of Panzhihua Iron and Steel (Group) Company. Occup Health. (2016) 32:881–4.

[3] Penson A Van Deuren S Worm-Smeitink M Bronkhorst E van den Hoogen FHJ van Engelen BGM . Short fatigue questionnaire: screening for severe fatigue. J Psychosom Res. (2020) 137:110229. doi: 10.1016/j.jpsychores.2020.110229, 32890861

[4] Gander P O'keeffe K Santos-Fernandez E Huntington A Walker L Willis J. Fatigue and nurses' work patterns: an online questionnaire survey. Int J Nurs Stud. (2019) 98:67–74. doi: 10.1016/j.ijnurstu.2019.06.011, 31319337

[5] Li MY Huang KH Hu YL Zhang YY Zhang YQ. Prevalence and influencing factors of occupational fatigue among nurses. Chin J Nurs. (2022) 57:853–8. doi: 10.3761/j.issn.0254-1769.2022.07.014

[6] Farag A Scott LD Perkhounkova Y Saeidzadeh S Hein M. A human factors approach to evaluate predicators of acute care nurse occupational fatigue. Appl Ergon. (2022) 100:103647. doi: 10.1016/j.apergo.2021.103647, 34837749

[7] Tanaka M Watanabe Y. Supraspinal regulation of physical fatigue. Neurosci Biobehav Rev. (2012) 36:727–34. doi: 10.1016/j.neubiorev.2011.10.004, 22040772

[8] Li XY Bao L Zhang YQ. Qualitative study on turnover reasons of nurses in tertiary hospitals of Shanghai. Nurs J Chin Peoples Liber Army. (2020) 37:42–5. doi: 10.3969/j.issn.1008-9993.2020.03.012

[9] Yang TT Li XY Yu LP Li YL Li SY Yan H. Mediating effect of work-family conflict between job stress and turnover intention in nurses. Nurs Res (China). (2020) 34:420–42. doi: 10.12102/j.issn.1009-6493.2020.03.015

[10] Zhang X Dong JQ Xu SH Li SY. Effects of shift work on circadian rhythm and fatigue among female ICU nurses. Chin J Nurs. (2021) 56:409–14. doi: 10.3761/j.issn.0254-1769.2021.03.016

[11] Chen HY Wu LH. Current status and correlation analysis of occupational fatigue and social support among orthopedic nurses. Occup Health. (2020) 36:442–444, 448.

[12] Johnson JV Hall EM. Job strain, work place social support, and cardiovascular disease: a cross-sectional study of a random sample of the Swedish working population. Am J Public Health. (1988) 78:1336–42. doi: 10.2105/ajph.78.10.1336, 3421392 PMC1349434

[13] Clough BA March S Chan RJ Casey LM Phillips R Ireland MJ. Psychosocial interventions for managing occupational stress and burnout among medical doctors: a systematic review. Syst Rev. (2017) 6:144. doi: 10.1186/s13643-017-0526-3, 28716112 PMC5514490

[14] Demir Y Dağ E Nal B Nal M Bektaş G. Determinants of nurses' job performance: the interaction of perceived occupational stress, psychological safety, and digital fatigue. BMC Nurs. (2025) 24:1443. doi: 10.1186/s12912-025-04055-z, 41291664 PMC12648931

[15] Lourenção LG Silva FGD Penha JGM Galvão DM Nascimento VF Sasaki NSGM d S . Interactions between occupational stress, compassion fatigue, and work engagement among nurses. Rev Bras Enferm. (2025) 78:e20240555. doi: 10.1590/0034-7167-2024-0555, 41221954 PMC12594483

[16] Niu L Zhang LX Mei SL He BW Guo JH. Analysis on fatigue level, stressors and sense of control of operating room nurses. J Changchun Univ Tradit Chin Med. (2016) 32:813–5. doi: 10.13463/j.cnki.cczyy.2016.04.056

[17] Steege LM Pinekenstein BJ Rainbow JG Arsenault Knudsen É. Addressing occupational fatigue in nurses: current state of fatigue risk management in hospitals, part 1. J Nurs Adm. (2017) 47:426–33. doi: 10.1097/NNA.0000000000000519, 28796024

[18] Long Y Zhang LN Ren DM Tian CM. Correlation analysis of intention to stay with job stressors, burnout, and work-family conflict among operating room nurses. Chin J Pract Nurs. (2020) 36:303–7. doi: 10.3760/cma.j.issn.1672-7088.2020.04.014

[19] Ding XT Li HP Yang YJ Su D Zhang T Xiao T. Analysis of nurses’ job control and its influencing factors. Anhui Med Pharm J. (2019) 23:1365–8. doi: 10.3969/j.issn.1009-6469.2019.07.023

[20] Barry S Leifels K Walter R Rahman A. Silent strength: the social support of mental health nurses. Int J Ment Health Nurs. (2025) 34:e70132. doi: 10.1111/inm.70132, 40905423 PMC12409769

[21] Najafi B Nasiri A. Support experiences for novice nurses in the workplace: a qualitative analysis. SAGE Open Nurs. (2023) 9:169212. doi: 10.1177/23779608231169212, 37187548 PMC10176594

[22] Wu C Lang HJ Zhang LF Zhang LY. Fatigue status and its influencing factors among emergency department nurses in tertiary hospitals in Xi’an. Occup Health. (2020) 36:580–4.

[23] Zhu HM Xiong YH. Correlation among job stressors, occupational fatigue and social support in nurses. China Contin Med Educ. (2019) 11:194–6. doi: 10.3969/j.issn.1674-9308.2019.01.090

[24] Adriaenssens J De Gucht V Maes S. Causes and consequences of occupational stress in emergency nurses, a longitudinal study. J Nurs Manag. (2015) 23:346–58. doi: 10.1111/jonm.12138, 24330154

[25] Wolf EJ Harrington KM Clark SL Miller MW. Sample size requirements for structural equation models: an evaluation of power, bias, and solution propriety. Educ Psychol Meas. (2013) 76:913–34. doi: 10.1177/0013164413495237, 25705052 PMC4334479

[26] Fang JB Qiu CJ. Occupational fatigue and its influencing factors among nurses. J Nurs Sci. (2009) 24:1–4. doi: 10.3870/hlxzz.2009.01.001

[27] Cohen S Kamarck T Mermelstein R. A global measure of perceived stress. J Health Soc Behav. (1983) 24:385–96. doi: 10.2307/2136404 6668417

[28] Wang Z Wang Y Wu ZG Chen DD Chen J Xiao ZP. Reliability and validity of the Chinese version of the perceived stress scale. J Shanghai Jiao Tong Univ. (2015) 35:1448–51. doi: 10.3969/j.issn.1674-8115.2015.10.004

[29] Li J Yang W Liu P Liu P Xu Z Cho SI. Psychometric evaluation of the Chinese (mainland) version of job content questionnaire: a study in university hospitals. Ind Health. 42:260–7. doi: 10.2486/indhealth.42.260, 15128178

[30] Wolf MG Mcneish D. Dynamic: an R package for deriving dynamic fit index cutoffs for factor analysis. Multivar Behav Res. (2023) 58:189–94. doi: 10.1080/00273171.2022.2163476, 36787513

[31] Hazzard B Johnson K Dordunoo D Klein T Russell B Walkowiak P. Work- and nonwork-related factors associated with PACU nurses' fatigue. J Perianesth Nurs. (2013) 28:201–9. doi: 10.1016/j.jopan.2012.06.010, 23886284

[32] Sagherian K Unick GJ Zhu S Derickson D Hinds PS Geiger‐Brown J. Acute fatigue predicts sickness absence in the workplace: a 1-year retrospective cohort study in paediatric nurses. J Adv Nurs. (2017) 73:2933–41. doi: 10.1111/jan.13357, 28586543

[33] Liu Y Wu LM Chou PL Wu L‐M Chou P‐L Chen M‐H . The influence of work-related fatigue, work conditions, and personal characteristics on intent to leave among new nurses. J Nurs Scholarsh. (2016) 48:66–73. doi: 10.1111/jnu.12181, 26641770

[34] Ikeda S Eguchi H Hiro H Mafune K Koga K Nishimura K . Work-family spillover, job demand, job control, and workplace social support affect the mental health of home-visit nursing staff. J UOEH. (2021) 43:51–60. doi: 10.7888/juoeh.43.51, 33678786

[35] Zhang Y Hu JX Wang N Xiong YS Duan BF. Mediating effects of vocational delay of gratification and job control on intrinsic motivation and work engagement of junior nurses in three grade-A tertiary hospitals in Kunming City. Occup Health. (2022) 38:782–7.

[36] Zhou XX Ye XC. Mediating effect of occupational stress between health-promoting behaviors and occupational quality of life in operating room nurses: a multicenter study. Mod Med. (2021) 49:675–9. doi: 10.3969/j.issn.1671-7562.2021.06.015

[37] Weng PP Lin YX Li ZZ. Analysis of influencing factors of occupational burnout and intervention suggestions among operating room nurses. China Med Sci. (2024) 14:119–23. doi: 10.20116/j.issn2095-0616.2024.04.28

[38] Woelfel I Wang T Pieper H Meara M Chen XP. Distortions in the balance between teaching and efficiency in the operating room. J Surg Res. (2023) 283:110–7. doi: 10.1016/j.jss.2022.10.032, 36402083

[39] Zhang Q Yu B Ou Y Zhou X Zou S Peng H . Progress of research on methods of human resource allocation in operating room nursing. Front Public Health. (2025) 13:1539108. doi: 10.3389/fpubh.2025.1539108, 40520322 PMC12164728

[40] Yu X Li M Du M Wang Y Liu Y Wang H. Exploring factors that affect nurse staffing: a descriptive qualitative study from nurse managers' perspective. BMC Nurs. (2024) 23:80. doi: 10.1186/s12912-024-01766-7, 38291385 PMC10829222

[41] Pi R Liu Y Yan R De Z Wan Y Chen Y . Nurses' occupational fatigue level and risk factors: a systematic review and meta-analysis. PLoS One. (2025) 20:e0326519. doi: 10.1371/journal.pone.0326519, 40680006 PMC12273991

[42] Hiestand S Waage S Forthun I Pallesen S Bjorvatn B. Factors leading to excessive fatigue in nurses - a three-year follow-up study. BMC Nurs. (2024) 23:446. doi: 10.1186/s12912-024-02066-w, 38951772 PMC11218166

[43] Jung FU Pabst A LöBNER M Luppa M Riedel-Heller SG. Behind the times? Associations of working-time autonomy with health-related and occupational outcomes in health care personnel- a latent profile analysis. BMC Public Health. (2024) 24:825. doi: 10.1186/s12889-024-18289-0, 38491458 PMC10943857

[44] Xu JB Zheng QX Jiang XM Zhuo Q Nian J-X Wang J-T. Mediating effects of social support, mental health between stress overload, fatigue and turnover intention among operating theatre nurses. BMC Nurs. (2023) 22:364. doi: 10.1186/s12912-023-01518-z, 37803427 PMC10559401

[45] Rahnfeld M Wendsche J Wegge J. Job demands and resources as drivers of exhaustion and leaving intentions: a prospective analysis with geriatric nurses. BMC Geriatr. (2023) 23:167. doi: 10.1186/s12877-023-03829-x, 36959574 PMC10037764

